# Non-communicable Diseases and Oral Health: An Overview

**DOI:** 10.3389/froh.2021.725460

**Published:** 2021-09-03

**Authors:** Thomas Gerhard Wolf, Maria Grazia Cagetti, Julian-Marcus Fisher, Gerhard Konrad Seeberger, Guglielmo Campus

**Affiliations:** ^1^Department of Restorative, Preventive and Pediatric Dentistry, University of Bern, Bern, Switzerland; ^2^Department of Periodontology and Operative Dentistry, University Medical Center of the Johannes Gutenberg University of Mainz, Mainz, Germany; ^3^Department of Biomedical, Surgical and Dental Sciences, University of Milan, Milan, Italy; ^4^Department of Oral Diagnostics, Digital Health and Health Services Research, Charité Universitätsmedizin Berlin, Berlin, Germany; ^5^FDI-World Dental Federation, Geneva, Switzerland; ^6^Department of Surgery, Microsurgery and Medicine Sciences, School of Dentistry, University of Sassari, Sassari, Italy; ^7^School of Dentistry, Sechenov First Moscow State Medical University, Moscow, Russia

**Keywords:** caries, non-communicable disease, NCD, oral health, periodontal disease, review

## Abstract

Non-communicable diseases (NCDs) such as cardiovascular and metabolic diseases, diabetes, cancer and diseases of the oral cavity such as caries or periodontitis represent a global and highly relevant problem due to demographic and epidemiological changes. NCDs are not only responsible for millions of deaths worldwide, but they cause relevant costs for national economies arise for the health care of societies. Assuming that oral health and general health are directly linked, emerging interactions between systemic and oral diseases are increasingly being researched. Common important risk factors have implications for economic, social, and moral determinants of health. Interdisciplinarity trained oral health professionals are needed to address the excessively high rates of inequities in oral health. The main reason that oral diseases are still a global health problem is related to mainly individual subjective high-risk approaches, which resulting in high costs and low effectiveness. A paradigm shift for a public health approach is needed at population level that integrates different health professionals who deal with NCDs. Oral care, like physical activity, is one of the most important lifestyle-related determinants of health. Widespread recognition of this kind of approach is critical to both reducing the impact of oral and non-oral NCDs. A multi-sectoral, comprehensive and integrated strategy is therefore necessary. The focus should be on social, environmental and population strategies, but should also support individual strategies.

## Introduction

Non-communicable diseases (NCDs) are a group of conditions related to modern lifestyle that can be explained by analyzing demographic and epidemiological transitions. They include diseases such as chronic respiratory diseases, dementia, diabetes, cardiovascular diseases, cancer, musculoskeletal and mental diseases, but also congenital or neurological diseases, genitourinary, blood and metabolic diseases, diseases of the skin, of sensory organs, of the digestive tract as well as of the oral cavity [1]. NCDs disproportionately affect people in low- and middle-income countries, where more than three quarters of global NCD deaths/year−32million—occur [2]. Furthermore, NCDs are characterized by a long disease phase and then, became the leading cause of death in our society [3].

The most prevalence oral diseases and disorders are linked to the four most prevalent non-communicable diseases such as cardiovascular disease (CVD), cancer, diabetes, and chronic respiratory diseases [4]. As consequence, oral health has been increasingly promoted as a part of the spectrum of the NCDs since the 2011 United Nations (UN) high-level meeting on NCDs.

Oral diseases and other systemic NCDs are connected by molecular and immunology-based evidences [5], since they share major common risk factors and commercial, moral and social determinants of health [6]. A common risk factor approach is therefore desirable and more rational compared to those disease specific. Since most chronic diseases have a multifactorial causation and are clustered in groups of people, an integrated action may be taken against risk factors related to different diseases; in addition, if one risk factor is common to several diseases, it can be attacked across disease boundaries. [7].

**Figure 1:**
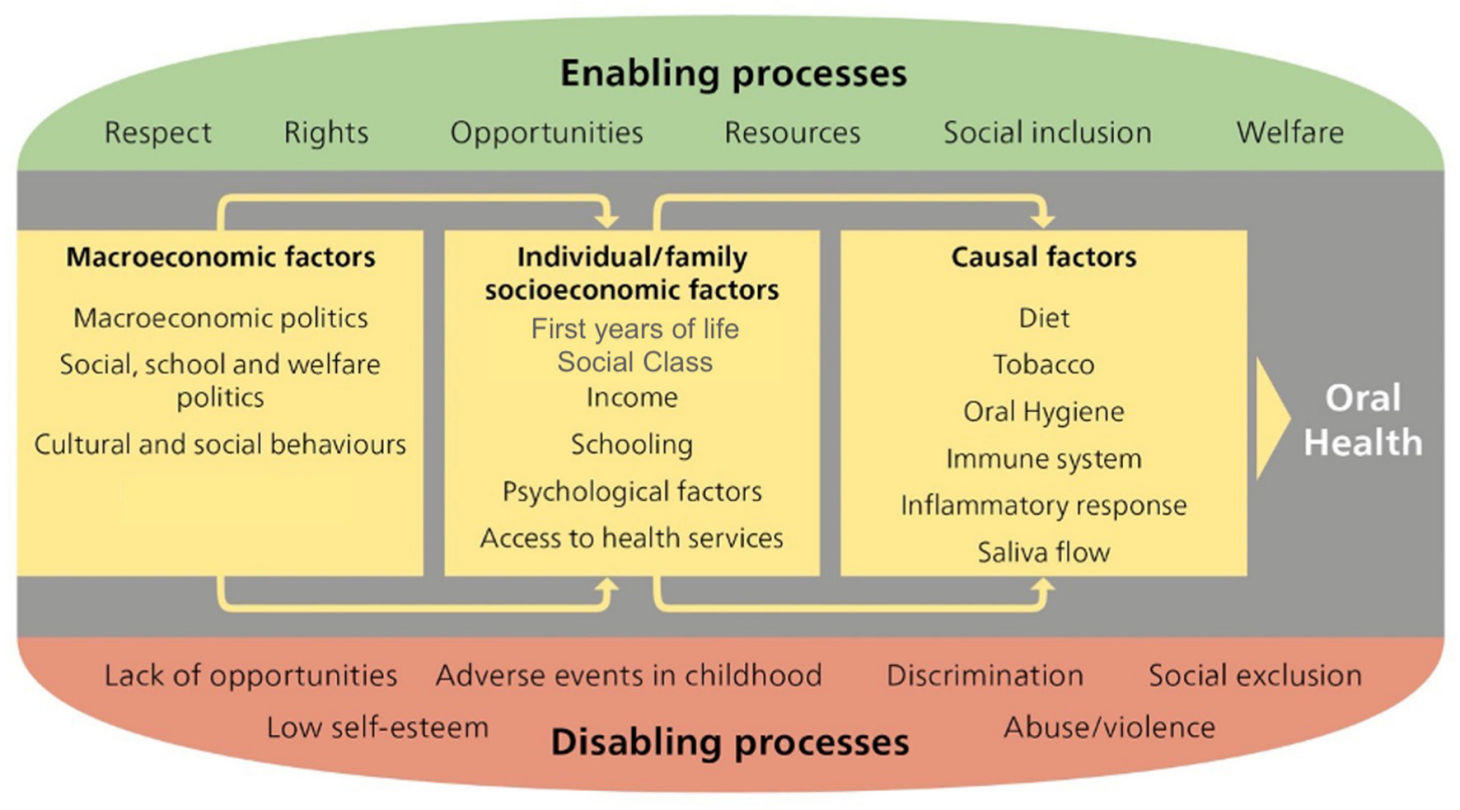
Oral health determinants [9].

Oral health, general health and well-being are intertwined and interactions between oral and systemic diseases evident [5]. Oral health is integral to overall health and shares many of the same risk factors as tobacco use, unhealthy diet consumption, physical inactivity and alcohol intake [8]. WHO highlights the need to focus not only on reducing disease, but also on tackling its root. This involves systematically addressing social, environmental and economic determinants of health [2] (Figure 1). Many of the social, environmental and economic determinants of health lie outside the health sector and can only be addressed by applying a multi-sectoral approach within policies and strategies for sustainable development. Therefore, promoting an integrated prevention approach and health promotion action is highly enticing. An improvement of public health level can be only achieved by reducing NCDs burden [9].

The recent definition of oral health proposed by the FDI World Dental Federation (FDI) quotes: “Oral health is multifaceted and includes the ability to speak, smile, smell, taste, touch, chew, swallow, and convey a range of emotions through facial expressions with confidence and without pain, discomfort, and disease of the craniofacial complex” [10]. A good oral health is a fundamental component of well-being and an essential element for the quality of life. Nevertheless, data from the Global Burden of Disease 2015 show that 3.5 billion people all over the word live with dental health related issues [3, 11].

Common oral diseases, especially periodontitis, interact with a variety of NCDs and different systemic disorders and conditions have the potential to affect a risk of developing oral diseases. An oral inflammation, as it happened during periodontitis process, can contribute to the systemic inflammatory burden and may effect on general health [12] as well as systemic diseases are related to a potential risk of complications in oral diseases [13]. The role of inflammatory pathway in periodontitis pathogenesis as a mediating mechanism in NCDs still needs to be elucidate [5, 12].

Despite being preventable, evidence shows that oral diseases are still among the most prevalent globally, with a significant impact on an individuals' quality of life, and high costs for Health Systems around the world [4]. Oral diseases such as dental caries and periodontal disease are the most prevalent oral pathologies, affecting 35 and 10% of the global population respectively [14, 15]. Oral cancer is among the top 15 most common cancers [16]. All these oral pathologies are included among NCDs.

Dental caries is a non-communicable, multifactorial, biofilm-mediated, diet modulated, dynamic disease, resulting in a net-mineral loss of dental hard tissues (enamel, dentine and cementum) [17–19] caused by acids produced by the cariogenic bacteria fermentation of free sugars. The process is dynamic, with periods of demineralisation and remineralisation of hard dental tissues with production of cavities, when demineralization wins over remineralization [17, 19]. If not arrested/treated, the process can cause pain, abscess and finally tooth lost. The main cause of caries is the diet rich in fermentable sugars [18]. Obesity and dental caries are well-known associated public health problems among children and adolescents worldwide, with income plays an important role on both conditions [20–23]. In children and adolescents, a positive correlation between Gross National Income (GNI) per capita and Body Mass Index was observed in 117 countries [22] The GNI per capita, the degree of inequality in a distribution of income (Gini coefficient) and the Unemployment rate were significantly correlated to caries lesions in Italian 12-year-olds, in which important differences in caries severity were found related to different socioeconomic backgrounds [23]. Distal factors such as socio-economic status, level of education, access to dental care services etc. act on the causal factors (diet, biofilm and host) of caries, by increasing or reducing their effects on the disease process [23, 24].

Periodontal disease is a chronic inflammatory condition affecting the tissues supporting the teeth [25]. It becomes as gingivitis, a reversible inflammation of gum tissues; in compromised immune response subjects, gingivitis might lead to periodontitis, causing destruction of periodontal ligament and alveolar bone with clinical attachment loss and presence of periodontal pockets. If not arrested/treated, the process can cause pain, abscess and, finally, tooth lost [26]. The main cause of periodontal disease is poor oral hygiene; however, as for caries, periodontal outcomes are influenced by social determinants with income and education level being the most influential variables [27]. While reports suggest that periodontal treatment is associated with improved health care outcomes and reduced costs [28, 29], an association between preventive dental care and improved health care outcomes has been observed, with an opposite association for extractions and endodontic treatments [30]. There is strong correlation between periodontitis, oral dysbiosis, and cardiovascular diseases as well as diabetes. Worsening of diabetes predisposes to poor oral hygiene as well as oral dysbiosis lead to an increased periodontal disease that creating a persistent chronic inflammatory response, which in turn may cause complications associated with diabetes [31].

Oral cancer includes cancer of the lips, tongue, gum, floor of mouth, palate, cheek mucosa, vestibule of the mouth and retromolar area. During 2007–2016, the incidence of cancer in these areas combined with pharynx increased [32]. When oral cancer is early diagnosed, the overall 5-year survival rate is about 85%, but if it has spread to surrounding tissues the rate decreases to around 67% and when cancer has reached distant part of the body, the overall rate drops to about 40% [33]. Main risk factors are tobacco use and alcohol consumption and HPV infection. High blood glucose level showed to be strongly associated with oral cancer, while overweight and obesity were found to be negatively associated; this association between lower BMI and oral cancer was found to be strong among tobacco users compared to former tobacco users and never users [34].

Conventional approaches to improvements of oral health have been shown to be ineffective and costly [8, 23, 24, 27, 35–37]. The economic burden is higher than the aggregate sum of direct costs (treatment costs), indirect costs (working/school days lost and economic productivity) and quality of life cost (i.e. pain, problems with biting, chewing and eating, tasting, speaking, and the expression of emotions such as smiling). Overall oral diseases accounted for more than 500 billion US$ [27, 38]. In the European Union oral diseases ranked third just behind cardiovascular diseases and diabetes. Oral diseases also have the effect of worsening the impact of other chronic diseases by increasing their economic weight, like the relationship between periodontal disease and diabetes and between caries and obesity [38, 39]. Integrated public health policies are needed in order to reduce/modify such risk factors. Nevertheless, this approach has been criticized [2, 40], since proximal risk factors alone capture only a part of the complex causative process that contributes to determine the health or disease status (lifestyles and social structure).

The situation is worse in low-income nations, where oral health diseases are often untreated as happen for caries with more than 50% lesions without cares [41]. In quantitative terms, oral and dental diseases affect more than 3 billion people worldwide (Figure 1). To date, the only comprehensive effort to estimate worldwide measures of population health, including oral health, by cause is the Global Burden of Diseases, Injuries, and Risk Factors (GBD) Study [40, 41]. A small number of high- and middle-income countries have undertaken regular national oral health surveys, but in most other countries very limited or no data on oral health status of their citizens are available [42].

This recognizes sharing of risk factors between NCDs and oral diseases, calls for a more efficient public health joint approach [27, 31–44]. A new generation of oral health indicators, able to measure the extent, magnitude and impact of oral conditions on individual quality of life, have been introduced to strengthen oral health population data, within an integrated surveillance and monitoring system at country level [45].

The WHO conceptual framework for action [46, 47] on the social determinants of health emphasizes the role of structural determinants of society, such as economic, social and welfare policies, in producing social inequalities. The social determinants of health (SDH) are the non-medical factors that influence health outcomes. SDH are the conditions in which people born, grow, work, live, and age. These conditions are produced by economic policies, social policies and political systems. The social determinants can be more important than health care or lifestyle choices in influencing health. Health and illness follow a social gradient with substantial inequalities in both oral and general health [47]. WHO has proposed three broad approaches to reducing health inequities produced by SDH: (a) targeted programmes for disadvantaged populations; (b) closing health gaps between worse-off and better-off groups and (c) addressing the social health gradient across the whole population. The development of a policy framework, as those proposed by the WHO, can help analysts and policymakers to identify levels of intervention and entry points for action on SDH, reducing inequities in the consequences of ill health suffered by different social groups.

The nature of oral diseases reflects both the persistence of socioeconomic inequalities and the limitations of care delivery systems that are not well designed to provide equitable access to health-sustaining resources. The high prevalence and social patterning of untreated oral diseases document the limitations of oral health prevention and disease management strategies and make the case that alternative approaches are needed desperately.

### The WHO Global infoBase and the Stepwise Approach to Surveillance (STEPS)

To predict the future burden of chronic disease, including oral disease, data collection and reporting standards are needed to ensure that the data can be effectively used to inform policy, and to plan preventive and surveillance activities for health. The WHO Global InfoBase stores the country data being collected as part of the STEPS approach; moreover, the data entered may also derive from a range of sources such as reports published in the literature or issued by different Ministry of Health.

### Health Care Systems

Historically, national dental care systems were dedicated on treatment of moderate or advanced diseases (reactive surgical interventions). Dental care systems were and still are structured mainly around acute care service delivery, when, in fact, the effective prevention and management of most dental disease requires a long-term care approach. In addition, populations oral health needs are often not aligned with availability, location, and dental care provided by public services.

In high- and middle-income countries, young children from low-income families, people with disabilities, institutionally elderly people, are often excluded from dental services, while healthy adults might be receiving unnecessary treatments [40, 41]. Such an approach cannot successfully face the global burden of oral disease. Moreover, compared with others health care professions, dental services are rarely integrated within the different health care systems. A shift to integrated models of oral care could help expand the role of oral health professionals and to collaborate with other health professionals on shared objectives. In the past, little emphasis was focused on health promotion or early intervention with few policy activities directed at social determinants; all the efforts were placed on traditional surgical dental practices. As outcome, dental workforce seldom includes people trained or incentivized to engage in integrated population-wide health promotion. An oral health workforce that includes staff trained to work across disciplines and with the skills to intervene upstream would significantly increase the capacity of the oral health sector to address the factors that have led to high rates of oral disease and inequalities [2, 42, 43]. A main reason why oral diseases are still a global health problem is linking to subjective high-risk approaches diverting so financial resources to effective primary preventive policies. The traditional approach based on high-risk individual treatment is costly and with a weak effectiveness.

## Conclusions

A paradigm shift is proposed and needed for a whole population public health approach that high- lights integration with other health care professionals tackling NCDs. Like physical activity for general health, oral care is one of the most important lifestyle-related determinants of health. Widespread recognition of these roles is critical to both reducing the impact of physical inactivity or failure to brush teeth on the risk of developing a range of systemic or chronic diseases. People should be motivated from birth to healthy behaviors such as regular physically active practice and oral hygiene maintenance through tooth brushing. In addition to the traditional lifestyle approach, including changing habits and behaviors to improve health, approaches focused on different strategies to facilitate the promotion of oral health, such as life course analysis, the salutogenic model, and social capital, are also needed in order to reduce inequities in general and oral health. To reach this goal, a multi-sectoral, comprehensive and integrated approach is needed. Based on the best available evidence and practices, these measures need to be implemented. Future researches with the aim of deeply understanding the system dynamics of the interplay of NCDs and oral diseases are desirable and necessary in order to plan and carried out social, political and environmental strategies to reduce the NCD-oral disease burden.

## Author Contributions

TGW, MGC, J-MF, GKS, and GC collected the various studies. All authors revised the article and approved the submitted version.

## Conflict of Interest

The authors declare that the research was conducted in the absence of any commercial or financial relationships that could be construed as a potential conflict of interest.

## Publisher's Note

All claims expressed in this article are solely those of the authors and do not necessarily represent those of their affiliated organizations, or those of the publisher, the editors and the reviewers. Any product that may be evaluated in this article, or claim that may be made by its manufacturer, is not guaranteed or endorsed by the publisher.
